# Treatment of Yellow Fever

**Published:** 1881-06

**Authors:** 


TREATMENT OF YELLOW FEVER.—Dr. Richard H. Day, of New Orleans, read a paper before the recent meeting of the Louisiana State Medical Society on the "Treatment of Yellow Fever" which we can endorse in every particular. During two epidemics we pursued a very similar course, and with almost the precise remedies he used in treating the *symptoms* of the dreaded disease rendered it as tractable, when seen sufficiently early, as any form of zymotic fever.

We hope to be able to have something to say on the subject of yellow fever ere long, and will then revert to Dr. Day's treatment again.

